# Escape of surgical smoke particles, comparing conventional and valveless trocar systems

**DOI:** 10.1007/s00464-023-10444-9

**Published:** 2023-10-04

**Authors:** Daniel Robertson, Frank Sterke, Willem van Weteringen, Alberto Arezzo, Yoav Mintz, The Technology Committee of the European Association for Endoscopic Surgery (EAES), Luigi Boni, Ludovica Baldari, Manish Chand, Hans Fuchs, Fanny Ficuciello, Stefania Marconi, George Mylonas, Young Woo Kim, Kiyokazu Nakajima, Marlies Schijven, Pietro Valdastri, Chen Sagiv, Pietro Mascagni, Piotr Myśliwiec, Wanda Petz, Francisco Sánchez-Margallo, Tim Horeman

**Affiliations:** 1 Department of Biomechanical Engineering, Faculty of Mechanical Engineering, Delft University of Technology, Mekelweg 2, 2628 CD Delft, The Netherlands; 2 Department of Pediatric Surgery, Erasmus MC Sophia Children’s Hospital, University Medical Center Rotterdam, Rotterdam, The Netherlands; 3 Department of Anesthesiology, Erasmus MC, University Medical Center Rotterdam, Rotterdam, The Netherlands; 4 Department of Surgical Sciences, University of Torino, Turin, Italy; 5 Department of General Surgery, Hadassah Hebrew University Medical Center, Jerusalem, Israel

**Keywords:** Surgical smoke, Valveless insufflation, Insufflation pressure, Laparoscopy

## Abstract

**Background:**

During minimal access surgery, surgical smoke is produced which can potentially be inhaled by the surgical team, leading to several health risks. This smoke can escape from the abdominal cavity into the operating room due to trocar leakage. The trocars and insufflator that are used during surgery influence gas leakage. Therefore, this study compares particle escape from a valveless (Conmed AirSeal iFS), and a conventional (Karl Storz Endoflator) system.

**Materials and methods:**

Using an in vitro model, a conventional and a valveless trocar system were compared. A protocol that simulated various surgical phases was defined to assess the surgical conditions and particle leakage. Insufflation pressures and instrument diameters were varied as these are known to affect gas leakage.

**Results:**

The conventional trocar leaked during two distinct phases. Removal of the obturator caused a sudden release of particles. During instrument insertion, an average of 211 (IQR 111) particles per second escaped when using the 5 mm diameter instrument. With the 10 mm instrument, 50 (IQR 13) particles per second were measured. With the conventional trocar, a higher abdominal pressure increased particle leakage. The valveless trocar demonstrated a continuously high particle release during all phases. After the obturator was removed, particle escape increased sharply. Particle escape decreased to 1276 (IQR 580) particles per second for the 5 mm instrument insertion, and 1084 (IQR 630) particles per second for 10 mm instrument insertion. With the valveless trocar system, a higher insufflation pressure lowered particle escape.

**Conclusions:**

This study shows that a valveless trocar system releases more particles into the operating room environment than a conventional trocar. During instrument insertion, the leakage through the valveless system is 6 to 20 times higher than the conventional system. With a valveless trocar, leakage decreases with increasing pressure. With both trocar types leakage depends on instrument diameter.

**Supplementary Information:**

The online version contains supplementary material available at 10.1007/s00464-023-10444-9.

During minimal access surgery, a trocar system is used to insufflate the abdominal cavity with carbon dioxide (CO_2_) gas to provide the surgeon with surgical workspace. This system is a combination of an insufflator, which regulates the flow and pressure of gas, and a trocar. Trocars are ports for surgical instruments to enter to the abdominal cavity, and seal the pressurized gas inside. Conventional trocar systems, also known as closed trocar systems, utilize valved trocars to prevent the outflow of insufflated CO_2_ gas.

In recent years, valveless trocar systems, also known as open, flow-through, constant-pressure barrier, gasketless, or valve-free systems have been developed. These use a pressure barrier within the trocar to maintain pneumoperitoneum pressure. This system was developed to overcome problems with conventional trocars, such as difficulty with the removal of specimens and needles, and soiling of the telescope lens [1–3]. Valveless systems inject CO_2_ at high flows through the working channel of the trocar to form the pressure barrier that maintains the pneumoperitoneum. The pneumoperitoneum pressure is measured at the distal trocar tip, while gas at the proximal side is taken up, filtered, and re-injected through the working channel [4].

Benefits of valveless trocars when compared to conventional trocars include a more constant insufflation pressure, less friction between the trocar and surgical instruments, and improved smoke removal [3, 5]. However, some controversy exists on the use of valveless trocar systems. Previous studies have shown that their use can lead to the entrainment of air [6, 7] which might affect peritoneal pressure, humidity, carbon dioxide concentration, and temperature [8].

During surgery, tissue is cut or coagulated using electrosurgical devices. This produces surgical smoke, which contains a mixture of water vapour, ultrafine particles, and vapourised biological materials [9]. Smoke can obscure the surgical field, leading to surgical errors, and longer operative times [10]. When smoke escapes from the abdominal cavity, it is prone to inhalation by the surgical team. A growing body of evidence on the health risks of exposure to surgical smoke includes respiratory and systemic infections, allergic reactions, and cancer [10]. Especially in times of Covid-19, surgeons became more aware of the risks related to surgical smoke as a potential carrier of harmful particles [11].

Various laparoscopic surgical smoke removal solutions have been developed to mitigate these risks and improve the overall surgical experience. These systems use filters, suction devices, and other methods to remove smoke generated during surgery. Conventional trocar systems commonly use a separate suction line to remove smoke. The valveless trocar systems adopt a more integrated solution to prevent surgical smoke particles from escaping into the operating room, which actively suctions and filters the abdominal gas before reinsufflating it.

Some studies characterized the particle interaction of trocar systems. No studies were found that quantified the number of particles that escape conventional trocar systems. For valveless trocar systems, Dalli et al. evaluated user safety and reported the escape of particle-rich aerosols through an AirSeal® Access Port (CONMED Corporation, Largo, USA) during transanal surgery [12]. Lathers et al. compared the intra-abdominal surgical smoke distribution between conventional and valveless trocar systems in a benchtop setup [13]. This study found a higher smoke evacuation rate when using valveless trocars, although a higher percentage of particles escaped from the trocar port. However, these studies do not allow for a direct comparison of the number of particles that escape these trocar systems.

This study investigates the difference in particle escape between a conventional and a valveless trocar system in an in vitro model. The model facilitates different pressure settings, and differently sized laparoscopic instruments, and contains a moving mechanical diaphragm to closely mimic the effect of ventilation on the pneumoperitoneum.

## Materials and methods

**Fig. 1:**
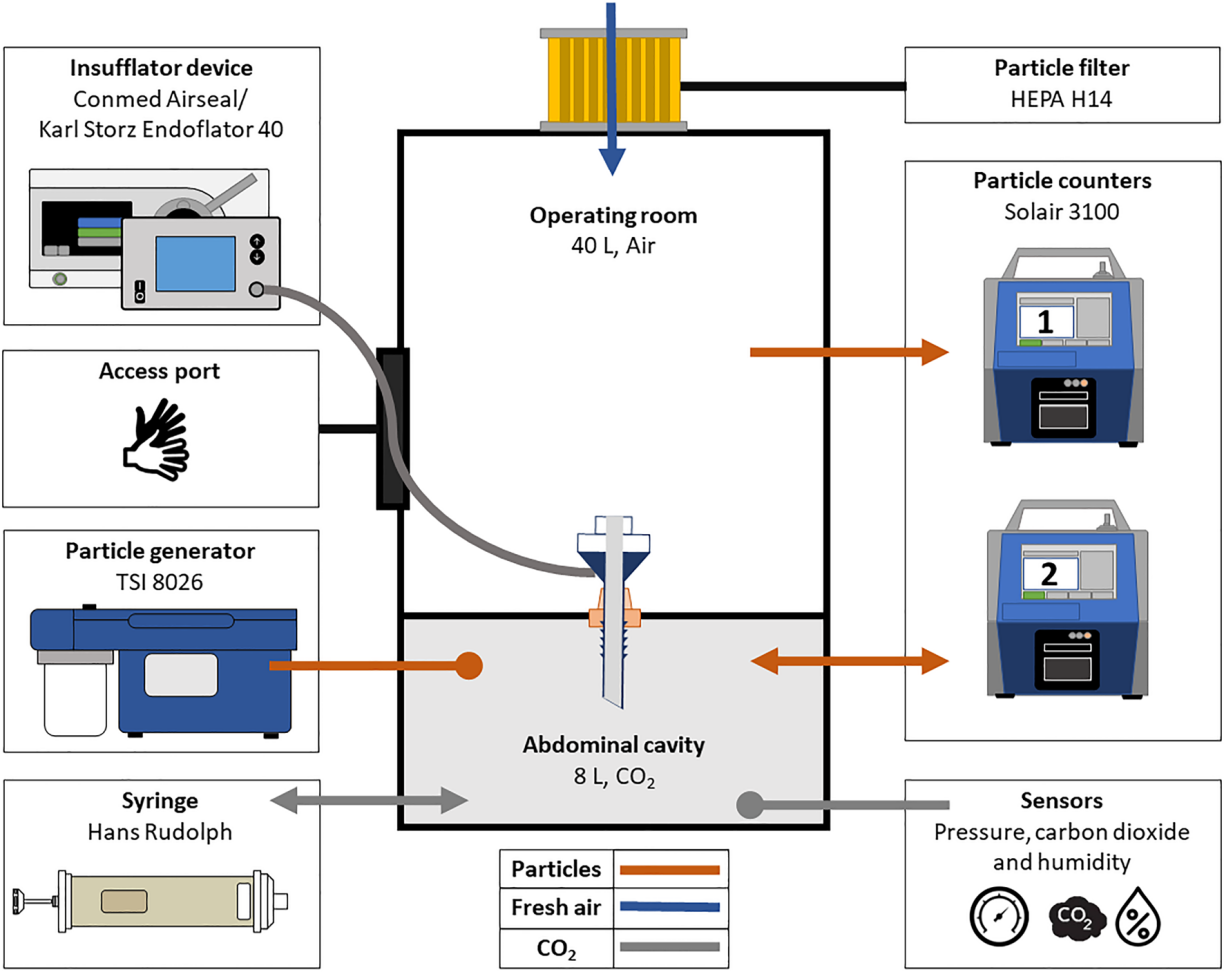
A schematic overview of the in vitro model for measuring particle leaks during laparoscopic surgery. The insufflator, access port, particle generator, and syringe are on the left side. On the right, filters and measurement equipment and HEPA filter

During this experiment, a conventional and a valveless trocar set were selected for comparison in an in vitro model. A protocol was defined to determine the influence of instrument diameter, pressure, and ventilation on particle leakage. An overview of the experimental setup is shown in Fig. 1. As the study did not involve human participants or animals, no approval from the Institutional Review Board (IRB) was required.

### Trocar systems

The conventional trocar system was a combination of an insufflator (ELECTRONIC ENDOFLATOR model 26 4305 20, Karl Storz GmbH & Co. KG, Tuttlingen, Germany) connected to a 12 mm trocar (Kii Optical Access System, Applied Medical Resources Corporation, Rancho Santa Margarita, CA, USA) using a 300 cm filtered insufflation tube (Insufflation tubing set with gas filter, model 031200-01, Karl Storz GmbH & Co. KG, Tuttlingen, Germany). The conventional trocar had an internal diameter of 13.4 mm at the proximal end and 13.1 at the distal end. The valveless trocar system included an AirSeal iFS insufflator (CONMED Corporation, Largo, USA), connected via a tri-lumen filtered tube set to the AirSeal Access port (ASM-EVAC1, CONMED Corporation, Largo, USA). The valveless trocar had an internal diameter of 13.4 mm proximally and 13.2 distally.

### In vitro model

The model consists of two circular acrylic reservoirs with a wall thickness of 4 mm, as can be seen in Fig. 1. The lower, 8 L, reservoir simulates the abdominal cavity and the upper, 40 L, reservoir serves as the operating room environment. The sensor equipment and smoke generator were placed within the abdominal cavity. This equipment occupies 2.5 L within the 8-L reservoir, resulting in an effective volume of 5.5 L, which is consistent with abdominal volumes found in literature [14]. The size of the operating room environment was chosen such that it represents the breathing space of the surgeon. A 0.2 µm HEPA H14 filter prevents background particles from moving in or out of the operating room environment. The filter also allows the pressure in the operating room environment to equalize to ambient pressure. The insufflator setting determines the pressure in the abdominal cavity.

The wall separating the reservoirs, holds a silicone nozzle through which the trocar can be inserted. To ensure repeatable measurement conditions, the silicone nozzle was designed to create an airtight seal between the trocar and abdomen. The airtightness of the seal was verified through a soap bubble test.

During surgery, mechanical ventilation influences the mechanics of the abdominal cavity through movement of the diaphragm. This is simulated with a 3 L syringe (Hans Rudolph Inc., Shawnee, USA) driven by a linear actuator (EGSL-BS-55-250-12.7P, Festo, Esslingen, Germany). The syringe was placed outside the abdominal cavity and could push gas back and forth to mimic the volume displacement of the diaphragm. As the model in this study has a stiffness different from the abdominal wall, the displacement of the syringe was chosen such that the pressure in the model mimics the abdominal pressure variations that occur during laparoscopic surgery. During this study, the total volume displacement was 30 mL at a simulated respiratory frequency of 15 breaths per minute. The diaphragm distortion in the model was similar to the pressure pattern described by Perretta et al. [7]. The actual displacements used for the study are added as Supplementary file 1.

To simulate surgical smoke, a particle generator (model 8026, TSI Incorporated, Shoreview, USA) was used to saturate the simulated abdominal cavity with particles. The particle generator was placed inside the abdominal cavity. This generator produces particles by evaporating a sodium chloride solution by pumping air through the solution at a rate of 1.5 L/min. The particles measured in this study ranged between 0.3 and 1.0 µm, which falls within the particle size range of surgical smoke [9, 15, 16].

### Protocol

#### Surgical intervention

During each recording, various surgical phases were simulated; each surgical phase was simulated for 60 s. The following phases were defined:

1. *Baseline* Start with the obturator inserted. For the valveless trocar system, the instructions for use indicate that the obturator should stay in during startup [17], the same was done when using the conventional insufflator system.
2. *Remove obturator* The obturator is removed such that an instrument can be inserted into the trocar.
3. *Diaphragm movement* The effect of diaphragm movement was investigated by activating the linear actuator and syringe.
4. *Insert instrument* For investigation of particle leak during normal use, an instrument was inserted through the entire trocar.
5. *Remove instrument* The instrument is removed to investigate the difference with phase four.
6. *Diaphragm movement off* Ventilation is turned off to investigate the difference with phase three.
7. *Baseline* The obturator is inserted to confirm the influence of the trocar on particle escape.

As the insufflation pressure and instrument diameter are known to affect gas leakage [18], the measurements were performed at three different insufflation pressures: 5, 10, and 15 mmHg, each with two instrument diameters. Therefore, four cases were defined: (1) conventional insufflation with a 5 mm instrument; (2) conventional insufflation with a 10 mm instrument; (3) valveless insufflation with a 5 mm instrument; and (4) valveless insufflation with a 10 mm instrument. Solid rods with the corresponding diameters were used as instruments. The conventional insufflator flow setting used for all cases was 5.0 L/min. The valveless insufflator was used in AirSeal mode, at 5.0 L/min insufflation flow, and low smoke evacuation.

Each recording was repeated three times to ensure repeatable conditions, for atmospheric changes due to the weather and conditions in the lab could affect the results. The pressure, humidity, carbon dioxide concentration, and temperature were monitored and recorded to verify the conditions within the abdominal cavity. In total, the combinations of pressures, instruments, and repetitions led to 18 recordings for each type of trocar system.

### Data collection and processing

Two laser particle counters were installed that measured particles ranging from 0.3 to 10 µm (Solair 3100, Lighthouse Worldwide Solutions, Medford, OR, USA). The first particle counter counted the particles in the operating room environment. The second particle counter was used to quantify the particles created within the abdominal cavity. A closed measurement system was required to allow pressure to build up in the abdominal cavity. To this end, the outlet of the particle counter fed back into the abdominal cavity. These devices count the number of particles that pass the sample port in three seconds at a flow rate of 28.3 L/min. Flow rates of all particle sizes were summed. By dividing the counted number of particles by three, the total number of particles per second were obtained.

The pressure sensor (ABPMRRN060MGAA5, Honeywell International Inc., Charlotte, NC, USA) measured pressure within the abdominal cavity relative to the ambient air. Humidity and temperature were measured using one sensor in the abdominal cavity (Asair AHT10, Guangzhou Aosong Electronic Co., Ltd., Guangzhou, China). Another sensor, placed inside the abdominal cavity, was used to record the carbon dioxide concentration, (STC31, Sensirion AG, Stäfa, Switzerland).

The data from the particle counters were retrieved over a serial connection. A custom data acquisition program was created in LabVIEW (NI Instruments Corp., Austin, TX, USA) to retrieve sensor data at a 1 Hz sampling frequency. The other sensors were read out through a LabJack T7 (LabJack Corporation, Lakewood, CO, USA), which was then recorded by the same data acquisition program. All data were automatically labelled and stored in tab-separated columned files.

The recordings were analysed using MATLAB (MathWorks, Inc., Natick, MA, USA). The analysis was limited by a small sample size. Therefore, instead of taking the mean, the median recording was calculated and presented. The spread between the measurements was reported by calculating the interquartile range (IQR).

All of the recordings were visually inspected to verify similar experimental conditions. Then, the variation of the experimental conditions between recordings of the valveless and conventional insufflation system was verified, by calculating the median and IQR of humidity, temperature, and carbon dioxide concentration for the combined 18 recordings, per phase. This verification of the experimental conditions was also performed for the combined three recordings of each pressure level, per trocar system. The effect of pressure, instrument diameter, and surgical phase on particle leakage was calculated by taking the median, minimum, and maximum particle leakage of three recordings. The average leakage per phase was calculated by taking the average over the three pressure levels, for each instrument diameter.

## Results

**Fig. 2:**
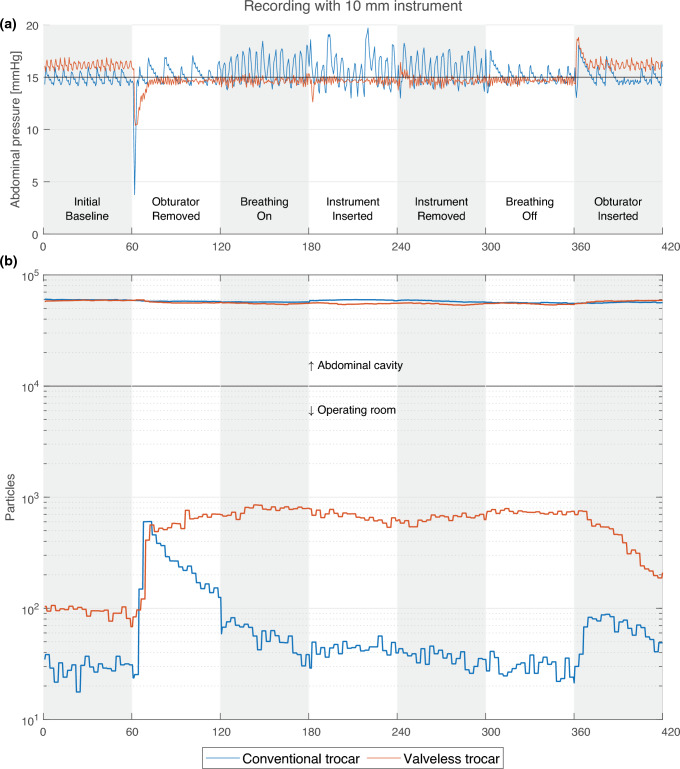
An example of two recorded samples in the conventional trocar system (blue) and the valveless trocar system (orange), both recordings were obtained using a 10 mm instrument while the insufflation pressure was set to 15 mmHg. **a** Pressure setting (black) and pressures measured within the abdominal cavity. **b** On the logarithmic *y*-axis, the counted number of particles, with a size ranging between 0.3 and 1 µm, within the operating room environment during every protocol phase (Color figure online)

During the measurements, the valveless trocar and the conventional trocar showed different responses to the protocol. To illustrate this, two recordings are shown in Fig. 2: one repetition for the valveless and conventional trocar systems at 15 mmHg with a 10 mm instrument. Figure 2a shows the pressure measured in the abdominal cavity using each of the trocar systems, and in Fig. 2b the number of particles in the operating room environment and in the abdominal cavity can be seen.

The first phase started with the obturator still inserted in the trocar. While the obturator was inserted, the pressure maintained by the valveless trocar was higher than the set pressure, whereas the conventional trocar showed no pressure difference. The variation seen in the first phase for both graphs was caused by the insufflation of gas to maintain the set abdominal pressure.

In phase two, after the obturator was removed, both trocars show a drop in abdominal pressure. The systems drop to 3.8 and 10.4 mmHg and recover to the set pressure in approximately 2.5 and 8 s, for respectively the conventional and valveless trocar system. After recovering the abdominal pressure, the pressure with the conventional trocar varied more (IQR of 1.2) than with the valveless trocar (IQR of 0.4) due to insufflation peaks. These peaks were not seen with the valveless system.

Diaphragm movement was started after the second phase. This can be seen in the graph of the conventional system, which varies in conjunction with the applied diaphragm movement. The valveless trocar did not respond as much to the diaphragm movement and maintained a more stable pressure (IQR of 0.4) than the conventional trocar (IQR of 2.2). The pressure maintained by the valveless trocar was consistently lower than the set pressure. During the last phase, the obturator was re-inserted, which can be seen in the pressure graphs for both systems.

The pressure response of each trocar system, as seen in Fig. 2a, can be related to the values in Table 1. The table shows the median and IQR pressures of six combined recordings of two instrument diameters. The median values and IQR per trocar system and phase were consistent throughout all recordings. During the phase in which the obturator was removed the valveless system had a lower median pressure when compared to the conventional system. During the diaphragm movement phases, the pressure IQR was higher for the conventional system when compared to the valveless system.

**Table 1:** Pressures recorded in mmHg within the abdominal cavity

Per pressure *n* = 6	Pressure (mmHg)	Baseline	Obturator removed	Breathing on	Instrument inserted	Instrument removed	Breathing off	Obturator inserted
Conventional	5	4.6 (1.0)	5.2 (0.9)	5.9 (1.5)	4.9 (1.4)	5.5 (1.0)	5.5 (0.2)	4.7 (0.2)
	10	9.8 (0.9)	10.0 (1.0)	11.7 (1.5)	10.0 (2.4)	10.9 (1.8)	9.9 (0.7)	9.8 (0.4)
	15	14.9 (0.8)	15.0 (0.7)	16.8 (1.6)	14.1 (1.6)	14.7 (1.2)	15.2 (0.7)	15.0 (1.0)
Valveless	5	6.2 (0.7)	4.6 (0.2)	4.6 (0.2)	4.7 (0.1)	4.6 (0.3)	4.7 (0.1)	5.8 (0.3)
	10	11.2 (0.8)	9.7 (0.4)	9.6 (0.3)	9.7 (0.3)	9.7 (0.2)	9.6 (0.2)	11.0 (0.5)
	15	16.1 (0.6)	14.7 (0.4)	14.9 (0.4)	14.7 (0.6)	14.5 (0.5)	14.8 (0.5)	16.0 (0.6)

Median and IQR for both trocar systems pressure for each phase

### Measurement conditions

Figure 2 is exemplary for all measurement conditions. To verify for consistency between measurement conditions, the humidity, temperature, particle number in the abdominal cavity, and CO_2_ level were monitored. Table 2 shows the medians and IQR’s of these values for the 18 recordings per trocar system, and similarity between the conditions that both trocar systems underwent.

**Table 2:** Medians and IQR for temperature, humidity, and CO_2_ recorded during each phase

Per system *n* = 18	Condition	Baseline	Obturator removed	Breathing on	Instrument inserted	Instrument removed	Breathing off	Obturator inserted
Conventional	Temp. (°C)	33.7 (2.3)	33.8 (2.2)	33.9 (2.1)	33.9 (2.0)	34.0 (2.0)	34.0 (2.1)	34.1 (2.0)
	Hum. (%)	26.1 (6.3)	26.1 (6.1)	26.2 (5.9)	26.1 (5.7)	26.2 (5.8)	26.4 (5.8)	26.5 (5.7)
	CO_2_ (%)	94.6 (0.8)	94.6 (0.7)	94.6 (0.8)	94.6 (0.7)	94.5 (0.7)	94.6 (0.7)	94.6 (0.6)
	Particles (10^4^)	6.2 (1.4)	6.3 (1.4)	6.3 (1.5)	6.2 (1.6)	6.2 (1.7)	6.3 (1.8)	6.2 (1.4)
Valveless	Temp. (°C)	33.0 (4.7)	33.0 (4.7)	33.0 (4.7)	33.1 (4.7)	33.2 (4.6)	33.4 (4.6)	33.6 (4.4)
	Hum. (%)	22.0 (2.8)	19.1 (2.2)	18.2 (2.2)	17.7 (2.1)	17.5 (2.2)	17.3 (2.2)	19.9 (2.3)
	CO_2_ (%)	96.5 (0.8)	96.9 (0.6)	97.0 (0.6)	97.0 (0.6)	97.0 (0.5)	97.1 (0.5)	96.8 (0.7)
	Particles (10^4^)	6.6 (0.9)	6.3 (1.0)	6.2 (1.1)	6.1 (1.0)	6.1 (1.2)	6.0 (1.2)	6.5 (1.3)

Within the abdominal cavity approximately 6 × 10^4^ particles were measured for the conventional and valueless trocar at 15 mmHg. The measurement conditions when using the conventional system remained constant. During recordings with the valveless system, the humidity level dropped slightly and the CO_2_ level increased slightly, which can be seen in Table 2.

### Particles

**Fig. 3:**
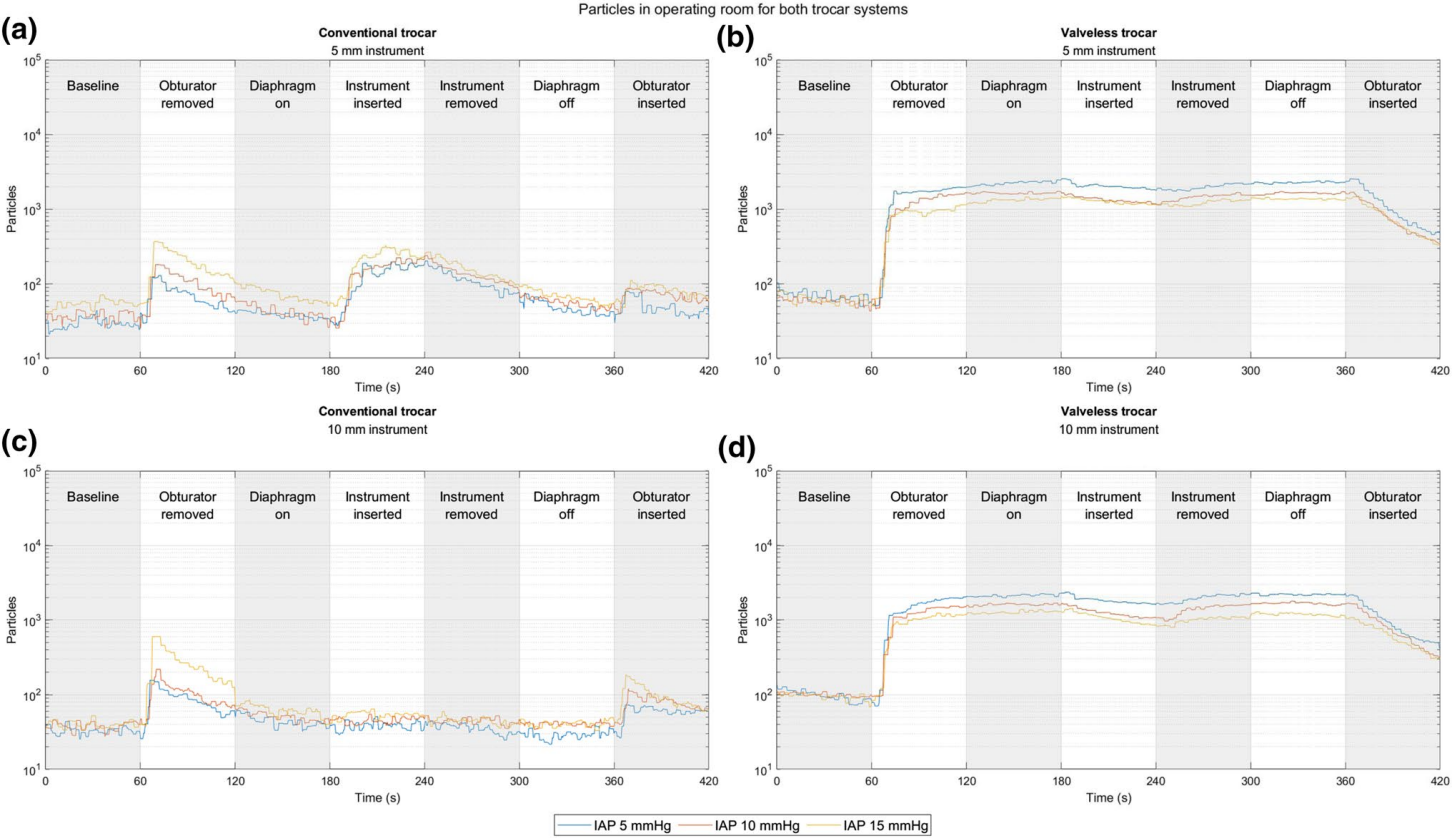
Four panels, with a logarithmic y-scale, showing the number of counted particles over time. Three different pressure conditions per panel: 5 mmHg (blue), 10 mmHg (orange), and 15mmHg (yellow). Included particle sizes, 0.3–1 µm. **a** Conventional insufflation and a 5 mm instrument. **b** Valveless insufflation and a 5 mm instrument. **c** Conventional insufflation and a 10 mm instrument. **d** Valveless insufflation and a 10 mm instrument (Color figure online)

Figure 3 shows the particles that escaped into the surgical workspace. Each subfigure shows a combination of a trocar system and instrument diameter. The median of three recordings for each pressure level is shown within each figure. The number of particles that escaped the trocar systems per second is shown in Table 3, the average per phase was calculated over the three pressure levels.

**Table 3:** Averaged exposure to particles for each phase and instrument, in particles per second

Per size *n* = 9	Instrument size (mm)	Baseline	Obturator removed	Breathing on	Instrument inserted	Instrument removed	Breathing off	Obturator inserted
Conventional	5	44 (27)	68 (50)	48 (33)	211 (111)	89 (21)	46 (17)	64 (22)
	10	40 (27)	73 (35)	38 (17)	50 (13)	35 (15)	39 (17)	61 (16)
Valveless	5	54 (70)	1716 (770)	1745 (700)	1276 (580)	1531 (640)	1722 (573)	396 (114)
	10	86 (44)	1508 (660)	1598 (670)	1084 (630)	1610 (771)	1637 (714)	358 (150)

When using the conventional trocar, a release of particles was seen after inserting or removing the obturator, corresponding with the pressure drop in Fig. 2a. The level of particles then decreased as the number of particles leaking into the surgical workspace was less than those removed by the particle counter. Figure 3a shows that after inserting the 5 mm instrument, the leakage of particles increased, which can be seen in the ‘instrument inserted’ phase. During the insertion phase of the 5 mm instrument, the average measurement for all pressure levels was 211 particles per second. Figure 3c shows that this leakage was absent when the 10 mm instrument was used, which had an average of 50 particles per second. When using the conventional trocar, a higher abdominal pressure led to a higher leakage of particles.

Figures 3b and d show the release of particles when the valveless system was in use. A sharp increase in particle leakage was seen when the obturator was removed from the valveless trocar, this also coincided with the pressure drop seen in Fig. 2a. The level of particles increased to a higher level, which remained relatively constant until the obturator was re-inserted. A slight decrease in particle release was seen when an instrument was inserted. The decrease was more substantial for the 10 mm instrument. During the instrument insertion phase, the average number of particles across the three pressure levels was 1276 and 1084 particles per second for the 5 mm and 10 mm instruments, respectively. A lower escape rate of particles was found when higher insufflation pressures were used.

## Discussion

This study investigated particle escape when using two different trocar systems in a benchtop setup. Each trocar system releases particles differently, depending on the set pressure and instrument diameter. This study shows that a higher number of particles is released into the surgical workspace when using a valveless trocar when compared to a conventional trocar.

The conventional trocar system releases particles in two distinct situations during surgery. The first concerns the insertion of a 5 mm instrument into a 12 mm trocar, which can be explained by the incomplete seal. This leakage is in line with a previous study by Robertson et al. that evaluated leakages in laparoscopic trocars [18]. The second moment of leakage occurs when the obturator is handled. The obturator provided with the conventional trocar has a hollow shaft with holes, providing a direct pathway for the abdominal gas to leak into the operating room environment.

The valveless trocar system has a tubeset which is designed to filter the abdominal gas before it is released into the surgical workspace. Similar to the study by Lathers et al. [13], this study shows that particles from the abdominal cavity are released from the trocar into the operating theatre despite the presence of filters.

Lower abdominal pressures in the valveless system caused more particles to escape into the operating room environment, which is also in line with the study by Lathers et al. [13]. This could be explained by the pressure barrier inside the trocar, providing a less efficient separation at lower pressures, causing more leakage into the operating room environment.

During use of the valveless trocar, the humidity and CO_2_ values deviated from their initial values. These variations were not observed when using the conventional trocar, and are likely due to the higher gas flow in the abdominal cavity of the valveless trocar. The humidity level within the in vitro model was not representative of a clinical setting. Therefore, the influence of the drop in humidity should be further investigated clinically. Although not the primary aim of this study, the valveless trocar system was observed to be better suited to mitigate pressure fluctuations in the abdomen due to mechanical ventilation than the conventional trocar system.

### Limitations and outlook

The in vitro model in this study was developed to create controllable conditions for comparison between the trocars, however, the influence of some factors will require further investigation. The influence of different steps of the protocol was different per trocar, per phase. Because these results were pronounced enough for a comparison between the trocars, the duration of the steps was not long enough for the conditions to return to baseline. In the future, by choosing a longer duration of the steps, it could give more insight into the behaviour of each system.

The number of detected particles in the upper volume is influenced by the placement of components, the extraction rate of the particle counters, and the size of the model. For this reason more studies are needed to determine the actual number of particles that a surgeon would breathe in to allow for a direct comparison. For example, such studies should include the effect of the ventilation systems within an OR.

Only one trocar of each type was used in the study to enhance reproducibility. Noteworthy is that not all conventional trocars follow the same design concept, leading to substantial differences in leak performance [18].

The model also differs from an in vivo setting as the stiffness of the model is different than the stiffness of an abdominal cavity. This might have altered the pressure effects. Additionally, the humidity level in the model was lower than in an in vivo situation. The influence of these factors on trocar performance still needs to be investigated.

When using the valveless trocar, the pressure in the abdomen showed significant fluctuations in several measurements when the obturator was inserted, which disappeared after obturator removal. The cause of these pressure variations could unfortunately not be determined. These conditions did not result in differing initial conditions between the two trocar systems.

Whether the particles that escape during laparoscopy enter the breathing air of operating room staff is not yet clearly understood. The actual exposure of surgery room staff to smoke particles has sparsely been studied [19], and should therefore be further investigated. Many studies have linked the inhalation of ultrafine particles, which are smaller than 0.1 µm, to neurological and psychiatric disorders [16]. These fine particles penetrate medical masks such as the standard N95 mask. This emphasizes the need to for adequate smoke removal during minimal access surgery, to prevent detrimental effects to either the patient or the surgical team.

## Conclusion

This study shows that valveless trocar systems release more particles into the operating room environment than conventional trocars for commonly used abdominal pressures. During instrument insertion, the leakage through the valveless trocar is 6 to 20 times higher than with a conventional trocar. Furthermore, the degree of this leakage depends on the set pressure and instrument size. For higher set pressures, the conventional trocar system shows a higher degree of leakage. The leakage from the valveless trocar is smaller with higher set pressures. Leakage also depends on instrument diameter, depending on the seal. Therefore, the choice for the surgeon to use the valveless trocar system has the advantage of having better pneumoperitoneal stability at the risk of increased exposure to potentially harmful smoke.

### Supplementary Information

Below is the link to the electronic supplementary material.

Supplementary file1 (XLSX 10 KB)
